# Metatranscriptomic analysis of colonic microbiota’s functional response to different dietary fibers in growing pigs

**DOI:** 10.1186/s42523-021-00108-1

**Published:** 2021-07-03

**Authors:** Jie Xu, Rongying Xu, Menglan Jia, Yong Su, Weiyun Zhu

**Affiliations:** 1 Laboratory of Gastrointestinal Microbiology, Jiangsu Key Laboratory of Gastrointestinal Nutrition and Animal Health, College of Animal Science and Technology, Nanjing Agricultural University, Nanjing, 210095 China; 2 National Center for International Research on Animal Gut Nutrition, Nanjing Agricultural University, Nanjing, 210095 China

**Keywords:** Carbohydrate active enzyme, Colonic microbiota, Dietary fiber, Metatranscriptomics, Pectin

## Abstract

**Background:**

Dietary fibers are widely considered to be beneficial to health as they produce nutrients through gut microbial fermentation while facilitating weight management and boosting gut health. To date, the gene expression profiles of the carbohydrate active enzymes (CAZymes) that respond to different types of fibers (raw potato starch, RPS; inulin, INU; pectin, PEC) in the gut microbes of pigs are not well understood. Therefore, we investigated the functional response of colonic microbiota to different dietary fibers in pigs through metatranscriptomic analysis.

**Results:**

The results showed that the microbial composition and CAZyme structure of the three experimental groups changed significantly compared with the control group (CON). Based on a comparative analysis with the control diet, RPS increased the abundance of *Parabacteroides*, *Ruminococcus*, *Faecalibacterium* and *Alloprevotella* but decreased *Sutterella*; INU increased the relative abundance of *Fusobacterium* and *Rhodococcus* but decreased *Bacillus*; and PEC increased the relative abundance of the *Streptococcus* and *Bacteroidetes* groups but decreased *Clostridium*, *Clostridioides*, *Intestinibacter*, *Gemmiger*, *Muribaculum* and *Vibrio*. The gene expression of CAZymes GH8, GH14, GH24, GH38, GT14, GT31, GT77 and GT91 downregulated but that of GH77, GH97, GT3, GT10 and GT27 upregulated in the RPS diet group; the gene expression of AA4, AA7, GH14, GH15, GH24, GH26, GH27, GH38, GH101, GT26, GT27 and GT38 downregulated in the INU group; and the gene expression of PL4, AA1, GT32, GH18, GH37, GH101 and GH112 downregulated but that of CE14, AA3, AA12, GH5, GH102 and GH103 upregulated in the PEC group. Compared with the RPS and INU groups, the composition of colonic microbiota in the PEC group exhibited more diverse changes with the variation of CAZymes and *Streptococcus* as the main contributor to CBM61, which greatly promoted the digestion of pectin.

**Conclusion:**

The results of this exploratory study provided a comprehensive overview of the effects of different fibers on nutrient digestibility, gut microbiota and CAZymes in pig colon, which will furnish new insights into the impacts of the use of dietary fibers on animal and human health.

**Supplementary Information:**

The online version contains supplementary material available at 10.1186/s42523-021-00108-1.

## Background

Dietary fibers are defined as the oligosaccharides, polysaccharides and derivatives that cannot be digested by the digestive enzymes into absorbable components in the small intestine but can be partly fermented by bacteria in the large intestine [1]. Common dietary fibers include resistant starch, soluble and insoluble fibers, as well as lignin [2]. As the dominant substrate for bacteria in pigs’ gastrointestinal tract, dietary fiber has been shown to enhance bacterial growth, resulting in higher faecal excretion of lipids, minerals and amino acids [3]. Dietary fibers are widely considered to be beneficial to health as they produce vitamins, short-chain fatty acids (notably butyrate) and other nutrients through microbial fermentation while facilitating weight management and boosting gut health [4]. Among the large number of genes that have been identified in the human gut microbiome, those that encode carbohydrate active enzymes (CAZymes) are of particular interest, as these enzymes are required to digest most of our complex repertoire of dietary polysaccharides [5]. Thus far, the gene expression profiles of CAZymes in the gut microbes of pigs are not well understood.

Three dietary fibers that differ in origin and composition are commonly used to improve gut health. Inulin is an effective prebiotic because it is resistant to digestion in the small intestine, but it can be fermented within the colon. It plays pivotal roles in adjusting the composition of intestinal microbiota, maintaining a normal intestinal environment, regulating intestinal function and improving human health. It has been demonstrated that inulin stimulates the growth of *Bifidobacteria* but limits the growth of potential pathogenic bacteria, such as *E. coli* and *Salmonella* [6]. Pectin is a soluble non-starch polysaccharide that is more fermentable in the hindgut than insoluble non-starch polysaccharides, producing short-chain fatty acids that are beneficial to the organism. Most of the glycoside hydrolase (GH) family enzymes are associated with pectin. Raw potato starch is a type of resistant starch that is also an insoluble dietary fiber. It is fermented by physiological bacteria in the large intestine to produce short-chain fatty acids and gases, stimulate the growth of beneficial bacteria and increase the number of *Bifidobacteria* [7]. Thus far, the mechanisms by which different types of fibers affect the microbiota metabolism in pigs’ colon remain unclear.

Metatranscriptomics is an efficacious method that can be utilised to predict the processes being mediated by microbes at a particular instant within an environmental sample, enabling the attainment of insight into the workings of microbial communities and potentially investigating their responses to environmental conditions [8]. In recent years, the application of metatranscriptomics in the analysis of microbial populations has increased extensively, but comparison results of the microbial functions’ response to different dietary fibers based on metatranscriptomics are very limited. Therefore, we conducted a metatranscriptomics study to test the hypothesis that the colonic microbial composition and gene expression of CAZymes are responsive to different dietary fibers in pigs.

## Results

### Overview of the metatranscriptomes

After removing the host-related sequences from clean reads, on the average, 100 million raw sequence reads were obtained from the metatranscriptome of each sample, and a total of 210.09 Gbp of high-quality sequences were generated from 16 samples after removing the adapters and quality filtering. The Q20 and Q30 base percentages of each sample were above 98.95 and 96.30%, respectively.

**Fig. 1:**
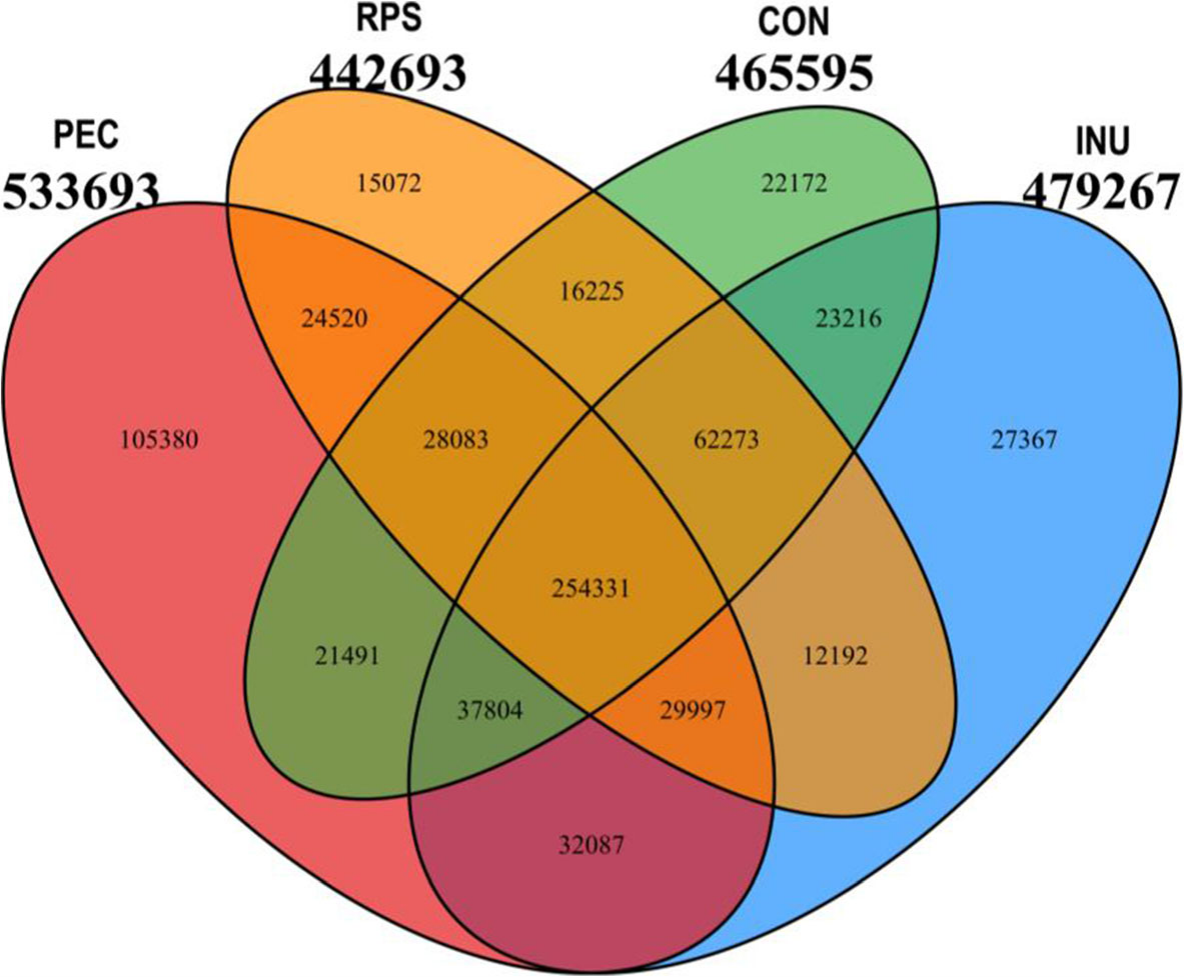
Species Venn analysis. Venn diagram showing the common and unique species in the colons of the pigs fed with the control (CON), inulin (INU), raw potato starch (RPS) or pectin (PEC) enriched diets

**Fig. 2:**
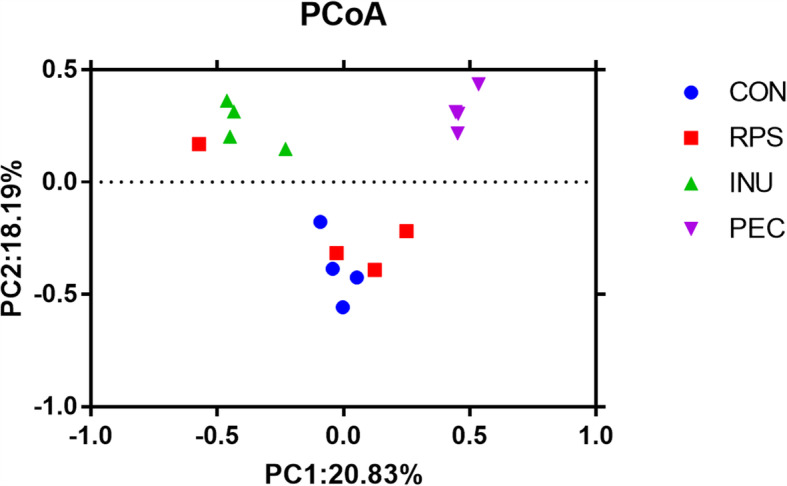
Bray-Curtis PCoA plot of colonic bacteria of the abundant genes. The percentages in the axis labels represent the percentages of variation explained by the principal components. A closer proximity of dots indicates higher similarity. CON, control diet; RPS, raw potato starch-enriched diet; INU, inulin-enriched diet; PEC, pectin-enriched diet

A total of 1,081,814 contigs were identified after de novo assembling using MEGAHIT in the 16 samples (four samples per group). The length of these contigs ranged from 546 to 1,004,658 bp, with an average length of 1623.05 bp. A total of 712,210 unigenes were clustered with CD-HIT (http://www.bioinformatics.org/cd-hit/) (95% identity and 90% coverage). Up to 254,331 core species were found in all four groups, and each group had its own unique species (Fig. 1). In general, the PEC group had the highest numbers of specific species compared with the other groups. Principal coordinate analysis (PCoA) based on Bray-Curtis distances showed that the colonic luminal digesta samples in the PEC group were clustered distinctly from those in the other groups, and INU samples was clearly segregated from control samples. While samples in the RPS group were more similar to controls, RPS-3 acting as an outlier, it is impossible to deny the influence of individual differences, but the main reason is the effect of RPS, as the other three samples in the RPS group are still relatively clustered (Fig. 2).

### Effect of different dietary fibers on colonic microbiota composition

**Fig. 3:**
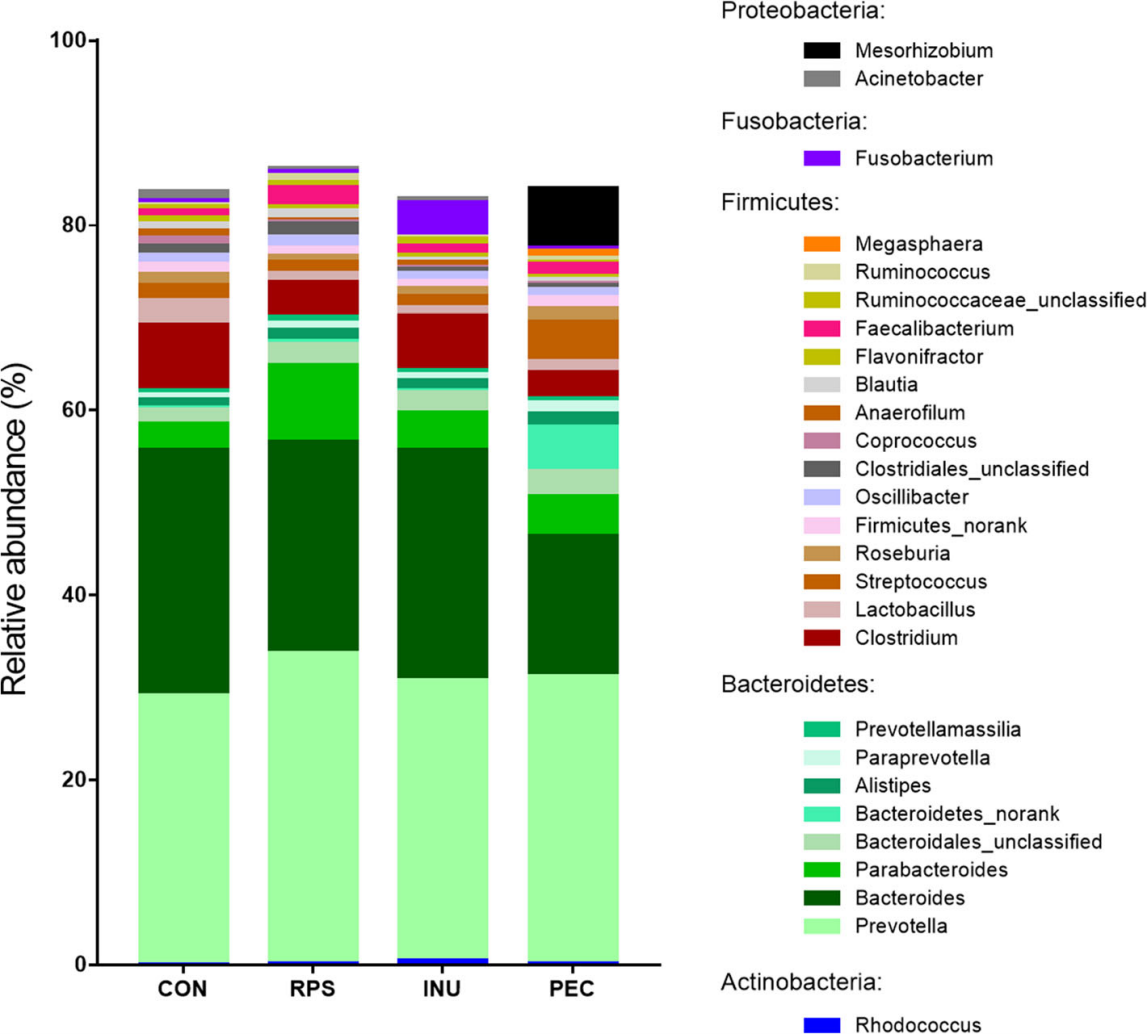
Distribution of dominant genera of bacteria (more than 0.5%) in at least one group. CON, control diet; RPS, raw potato starch-enriched diet; INU, inulin-enriched diet; PEC, pectin-enriched diet

The distribution of dominant bacteria in each group is shown in Fig. 3. In the CON group, the bacteria detected in the proximal colonic luminal digesta samples belonged to 55 different phyla. The most abundant phylum was *Bacteroidetes*, followed by *Firmicutes* and *Proteobacteria*. Up to 1002 bacteria genera were observed in this group, with *Prevotella*, *Bacteroides* and *Clostridium* as the most abundant. The INU group contained 968 genera belonging to 57 phyla, the RPS group had 49 phyla made up of 918 genera and the PEC group contained 60 phyla and 1131 genera.

At the phylum level, the abundance of *Verrucomicrobia* in the RPS group was lower (fold change > 2 or < 0.5; FDR < 0.05) than that in the CON group. The abundance of *Verrucomicrobia* in the INU group was also lower (fold change > 2 or < 0.5; FDR < 0.05) than that in the CON group, while the abundance of *Fusobacteria*, *Actinobacteria* and *Cyanobacteria* were greater (fold change > 2 or < 0.5; FDR < 0.05) than those in the CON group. Meanwhile, the populations of *Proteobacteria*, *Spirochaetes* and *Verrucomicrobia* phyla were greater (fold change > 2 or < 0.5; FDR < 0.05) in the PEC group than in the CON group colonic digesta samples (Additional File 1).

**Fig. 4:**
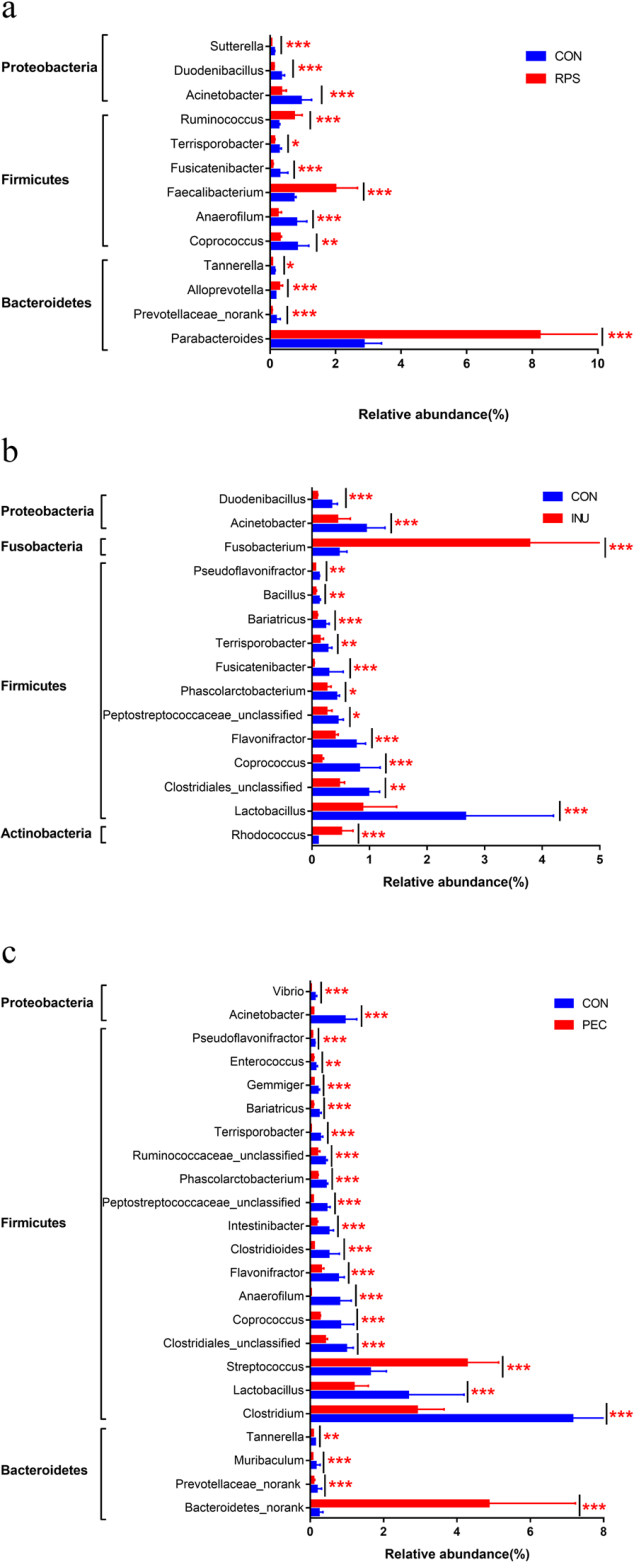
Relative abundances (percentage) of microbial genera significantly affected by RPS (**a**), INU (**b**) and PEC (**c**) in the pigs’ colon. CON, control diet; RPS, raw potato starch-enriched diet; INU, inulin-enriched diet; PEC, pectin-enriched diet. The FDR was calculated based on the *p*-value. *FDR < 0.05, **FDR < 0.01, ***FDR < 0.001

At the genus level, compared with the CON group, significant shifts were detected (*p* < 0.05) in 13 genera in the RPS group, while 15 and 23 genera changed significantly in the INU and PEC groups, respectively. (Fig. 4). The abundance of *Parabacteroides*, *Faecalibacterium*, *Ruminococcus* and *Alloprevotella* increased but *Sutterella* decreased in the RPS group. Inulin supplement increased the abundance of *Fusobacterium* and *Rhodococcus* but decreased Bacillus. The abundance of *Streptococcus* and *Bacteroidetes_norank* increased but *Clostridium*, *Clostridioides*, *Intestinibacter*, *Ruminococcaceae_unclassified*, *Gemmiger*, *Muribaculum*, *Enterococcus* and *Vibrio* decreased in the PEC group.

### Effect of different dietary fibers on the activities of colonic CAZymes

**Fig. 5:**
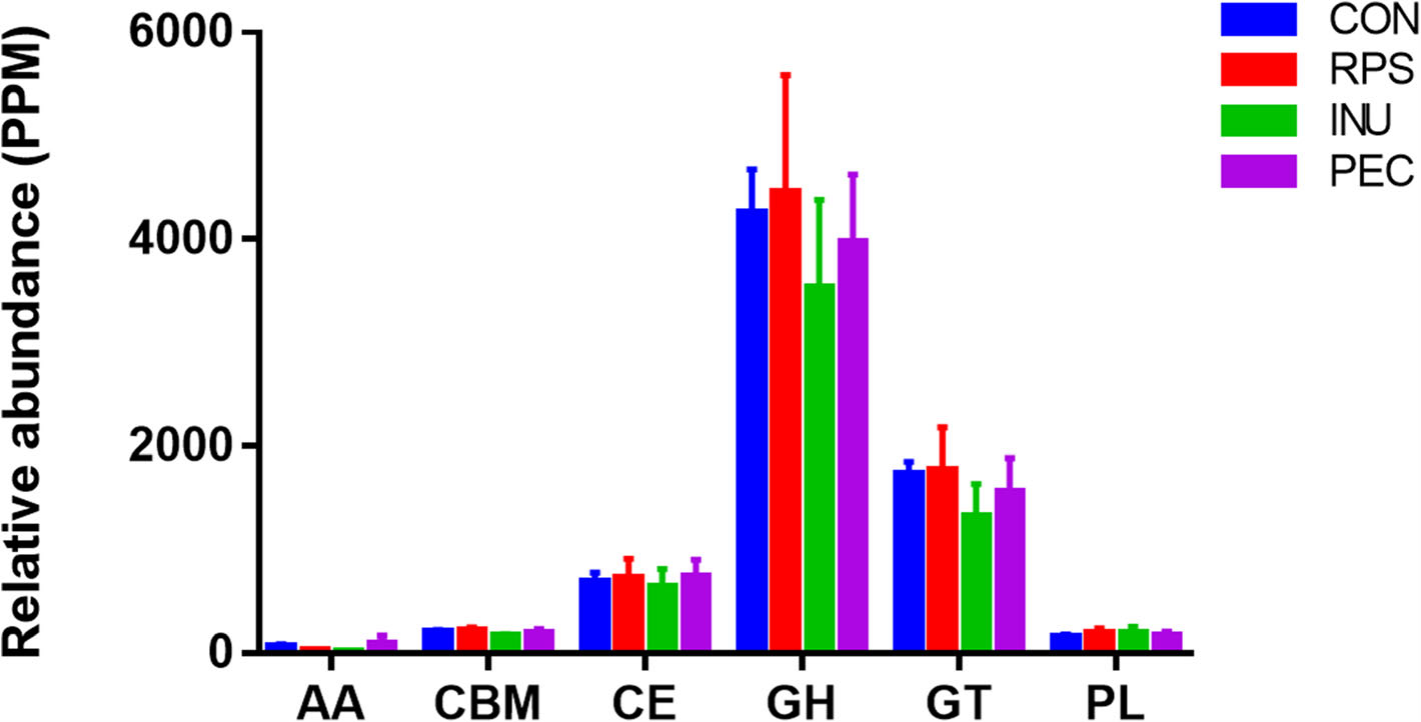
Distribution of CAZyme at the class level in each group. AA, auxiliary activities; CBM, carbohydrate-binding modules; CE, carbohydrate esterases; GH, glycoside hydrolases; GT, glycosyl transferases; PL, polysaccharide lyases. CON, control diet; RPS, raw potato starch-enriched diet; INU, inulin-enriched diet; PEC, pectin-enriched diet

In terms of CAZyme profiles, 222 CAZyme families were detected, including seven auxiliary activities (AAs), 36 carbohydrate-binding modules (CBMs), 15 carbohydrate esterases (CEs), 94 glycoside hydrolases (GHs), 57 glycosyl transferases (GTs) and 13 polysaccharide lyases (PLs). As shown in Fig. 5, GHs were the most abundant class in all four groups, but the distribution of CAZymes at the class level did not exhibit significant change among the four groups.

**Fig. 6:**
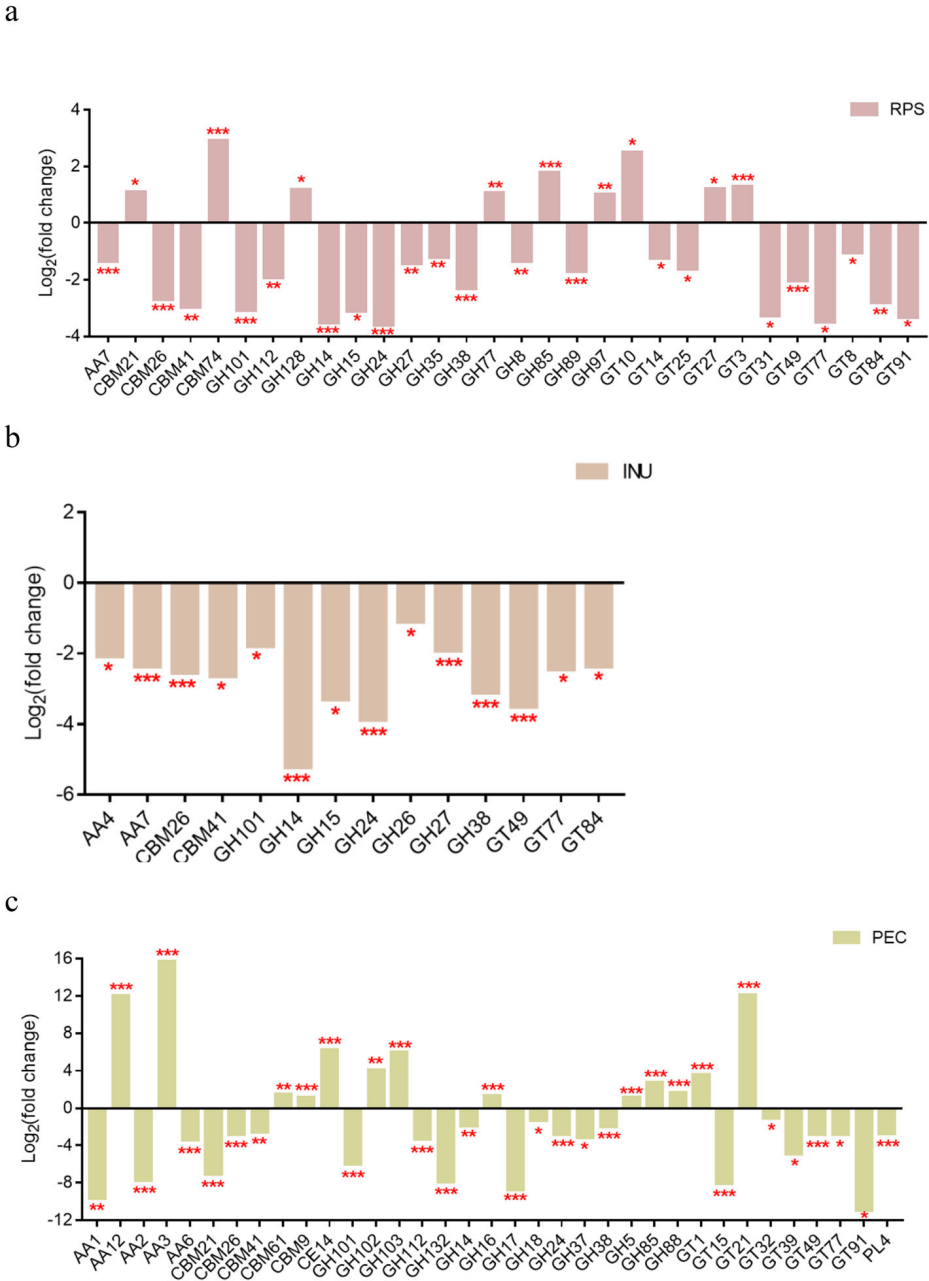
Gene expression of CAZymes significantly affected by RPS (**a**), INU (**b**) and PEC (**c**) in the pigs’ colon. The CAZyme families are classified according to the CAZy database. AA, auxiliary activities; CBM, carbohydrate-binding modules; CE, carbohydrate esterases; GH, glycoside hydrolases; GT, glycosyl transferases; PL, polysaccharide lyases. RPS, raw potato starch-enriched diet; INU, inulin-enriched diet; PEC, pectin-enriched diet. The values are presented as log_2_ (fold change). The FDR was calculated based on the *p*-value. *FDR < 0.05, **FDR < 0.01, ***FDR < 0.001

Compared with the CON group at the family level, some changes were found in the dietary fiber groups (Additional File 2). The gene expressions of the CAZymes that were significantly affected by the different dietary fibers in the pigs’ colon are shown in Fig. 6. Thirty CAZyme families changed significantly (fold change > 2 or < 0.5; FDR < 0.05) in the RPS group. The specific changes were as follows: nine CAZymes (CBM21, CBM74, GH128, GH77, GH85, GH97, GT10, GT27 and GT3) were upregulated in the mRNA expression while 21 CAZymes (AA7, CBM26, CBM41, GH101, GH112, GH14, GH15, GH24, GH27, GH35, GH38, GH8, GH89, GT14, GT25,GT31, GT49, GT77, GT8, GT84 and GT91) were downregulated. In the INU group, 14 CAZyme families, namely, AA4, AA7, CBM26, CBM41, GH101, GH14, GH15, GH24, GH26, GH27, GH38, GT49, GT77 and GT84 were downregulated significantly. Meanwhile, 35 CAZyme families changed significantly in the PEC group, 13 CAZymes (AA12, AA3, CBM61, CBM9, CE14, GH102, GH103, GH16, GH5, GH85,GH88, GT1 and GT21) manifested higher abundance while 22 CAZymes (AA1, AA2, AA6, CBM21, CBM26, CBM41, GH101, GH112, GH132, GH14, GH17, GH18, GH24, GH37, GH38, GT15, GT32, GT39, GT49, GT77, GT91 and PL4) were lower than in the CON group. Among the altered CAZyme families, four CAZymes, namely, AA7, GH15, GH27 and GT84, were downregulated in both INU and RPS groups. GH112 and GT91 decreased while GH85 increased in both RPS and PEC groups while CBM21 increased in the RPS group but decreased in the PEC group. Two altered CAZymes (AA4 and GH26) were specific in the INU group, 14 CAZymes (CBM74, GH128, GH35, GH77, GH8, GH89, GH97, GT10, GT14, GT25, GT27, GT3, GT31 and GT8) were specific in the RPS group and 23 CAZymes (AA1, AA12, AA2, AA3, AA6, CBM61, CBM9, CE14, GH102, GH103, GH132, GH16, GH17, GH18, GH37, GH5, GH88, GT1, GT15, GT21, GT32, GT39 and PL4) were specific in the PEC group.

### Correlation between CAZymes and colonic microbiota

One of the most critical roles of the microbiota is their ability to utilise complex carbohydrate sources. The network of correlation analyses between CAZyme classes and the microbiota (at the genus level) showed that *Prevotella* and *Tannerella* primarily contributed the CAZyme-encoding gene fragments of the GHs, the GTs were mainly produced by *Prevotellamassilia* and *Prevotella*, *Prevotellamassilia* and *Roseburia* primarily contributed to CBMs, *Lachnotalea* and *Butyricimonas* primarily contributed to CEs, *Butyricimonas* and *Mediterranea* primarily contributed to PLs and AAs were mainly produced by *Turicibacter* and *Chlamydia* in the growing pigs’ colon metatranscriptome among the significantly changed bacterial genera (Additional File 3).

**Fig. 7:**
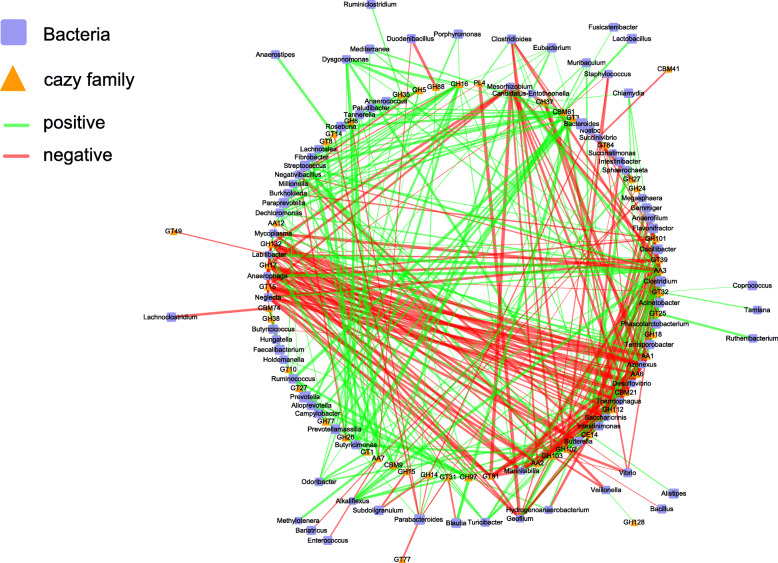
Network of correlations between the bacteria and CAZyme families that were changed significantly by the dietary fibers. The network is displayed graphically in the form of nodes (bacteria or CAZyme families) and lines (significant interactions among nodes). The Spearman correlation coefficient reveals the association between the changes in bacteria genera and the expression of the CAZyme family genes that changed significantly in at least one experimental group (SCC > 0.5 or < − 0.5 and *p* < 0.05). The lines’ colours represent two kinds of correlation: green for positive correlation and red for negative correlation

To explore the potential association between the microbiota and CAZymes, Spearman’s rank correlations were constructed between the 51 bacterial genera and 79 CAZyme families that were significantly affected by the three dietary fiber treatments. The results revealed a strong association with a threshold of Spearman’s correlation coefficient > 0.5 or < − 0.5 and *p* < 0.05. As shown in Fig. 7, the productions of CBM21 and GT91 were negatively correlated with the abundance of *Sutterella*. *Parabacteroides* had a negative correlation with the production of GH14, GH24, GH8, GT14, GT31 and GT77 but contributed proportions of GT10 and GT3. *Alloprevotella* and *Ruminococcus* contributed proportions of GH77, GH97, GT10, GT27 and GT3, while *Faecalibacterium* was a contributor of CBM74 and GH38. *Streptococcus*, *Clostridioides*, *Intestinibacter*, *Vibrio*, *Clostridium*, *Gemmiger*, *Muribaculum* and *Enterococcus* altered significantly specific to the PEC vs. CON group. *Streptococcus* had a positive correlation with the production of AA12, AA3, CBM61, GH102, GH103, GH16 and GH5. *Clostridioides* contributed proportions of AA1, CBM21 and PL4 but was negatively correlated with the production of AA3, CBM61, GH102 and GH103. *Intestinibacter* had a negative correlation with the production of AA3, CE14 and GH102. *Vibrio* had a negative correlation with the production of AA12, AA3, CBM61, GH102 and GH103. *Clostridium* was a contributor of GH18 and GT32 but was negatively correlated with the production of AA3. *Gemmiger* contributed proportions of GH101 and GH112 but was negatively correlated with the production of AA3 and CE14. *Muribaculum* was a contributor of CE14 and GH37.

**Fig. 8:**
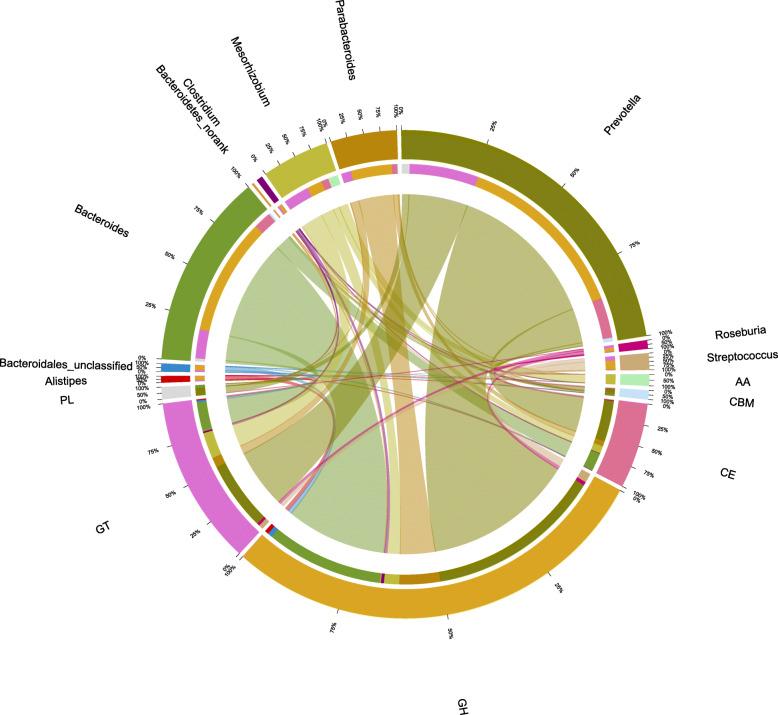
Taxonomic distribution (genus level) of the predicted CAZymes identified from the metatranscriptomes in the PEC group. The CAZyme families and the corresponding genus are shown on the sides above and below, respectively. The inner ring designates the total number of unigenes encoding a given CAZyme class (below) and the total number of CAZymes associated with the given genus, while the outermost ring designates the relative abundance of a given CAZyme family (below) and the relative abundance of unigenes from a given genus (above). The width of the bars between a given genus and a given CAZyme family indicates their relative abundance compared with that in other genera

**Fig. 9:**
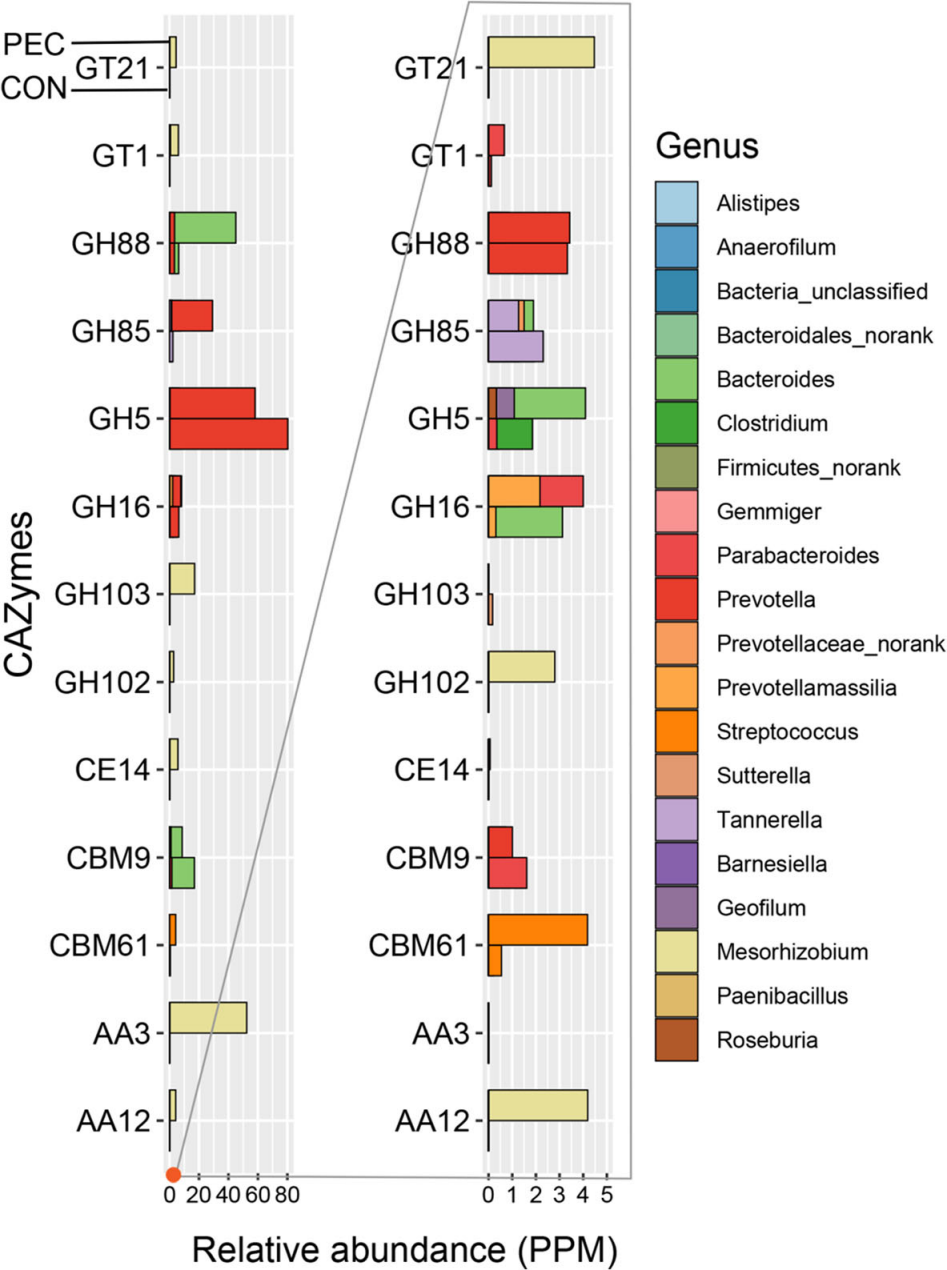
Contributions of CAZymes from the major microbial communities in the growing pigs’ colons (PEC vs. CON). The graphs show the abundance of the top 15 genera that constitute the major contributors of CAZymes to the growing pig colon ecosystem. AA, auxiliary activities; CBM, carbohydrate-binding modules; CE, carbohydrate esterases; GH, glycoside hydrolases; GT, glycosyl transferases. PEC, pectin-enriched diet; CON, control diet

According to the taxonomic distribution (top 10 genera) of the predicted CAZymes identified from the metatranscriptomes in the PEC group, *Prevotella*, *Bacteroides*, *Mesorhizobium* and *Parabacteroides* were the largest genera in the predicated AAs, GHs, GTs, CEs, PLs and CBMs. It is worth noting that *Streptococcus* was also the major microbial origin of the predicted CBMs (Fig. 8). Focusing on the contributions of the CAZymes from the major microbial communities in growing pigs’ colon in the PEC group compared with the CON group, it is worth noting that *Prevotella* was the main contributor of GH5, *Mesorhizobium* was the main contributor of GH16 and *Streptococcus* was the main contributor of CBM61 in the PEC group (Fig. 9).

## Discussion

In this study, in order to comprehensively compare the different dietary fibers’ effects on luminal microbiota composition and the activities of colonic CAZymes, we fed the pig specimens with three different fiber diets. Considerable microbiota variations occurred in the proximal colonic luminal digesta samples in the different dietary fiber groups. Compared with the INU and RPS groups, the PEC group was characterised by more colonic microbiota changes, suggesting that a pectin-enriched diet may have a more substantial impact on pigs’ colonic microbiota.

Our study showed that the average daily feed intake, final body weight and average daily gain were lower in the PEC group than in the other three groups, while the ratio of feed-to-gain in the PEC group was higher than in the other three groups [9]. Similar to previous studies [10, 11], INU and RPS did not affect the growth performance of the pigs within a certain quantity. Pectin diet could also reduce the feed intake and body weight of rats [12], probably because of its viscosity, bulking characteristic and water retention capacity. In terms of nutrient digestibility, there was no significant difference in crude fiber digestibility among the groups. A previous study also proved that crude fiber digestibility was not affected by inulin diet in growing castrated pigs [13]. RPS reduced (*p* < 0.05) the digestibility of crude protein, which is consistent with earlier studies [14, 15]. Previous research has shown that inulin supplementation improved the digestibility of crude fat, but it had no effect on the growth performance of broiler chickens [16]. Meanwhile, pectin supplementation decreased the apparent digestibility coefficients of crude fat [17], which is in agreement with our results. Although the negative effect of pectin on pig growth has been established, its impact on gut microbial function has not been fully clarified.

Significant (fold change > 2 or < 0.5; FDR < 0.05) increase was detected in *Parabacteroides*, *Ruminococcus*, *Faecalibacterium* and *Alloprevotella* in the RPS group, while a concurrent reduction was observed in *Sutterella.* The increase in *Faecalibacterium* can be associated with augmented RPS consumption [18] and its abundance is negatively correlated with crude protein digestibility (*p* < 0.05) [19]. In addition, a previous study showed that *Sutterella* increased substantially in the ceca of mice fed with raw potato starch [20]. In our study, we found that the RPS group showed a decrease in *Sutterella*, which was opposite the findings of the abovementioned study on mice. The disparity in the results may be related to the different experimental animals. The larger individual variations of pigs should also be considered. Inulin diet could increase the abundance of *Bifidobacteria* and *Faecalibacteria* while decreasing the abundance of *Bacteroides* [21–23]. In diabetic rat gut microbiota, inulin treatment upregulated the abundance of the probiotic bacteria *Lactobacillus* while downregulating the abundance of *Desulfovibrio*, which produce lipopolysaccharide [24]. However, these changes were not noted in our study. A possible reason is that the amount of inulin used in this study is different from that used in other studies. In our study, the abundance of *Fusobacterium* and *Rhodococcus* increased significantly, while *Bacillus* decreased specific to the INU group. Previous reports have suggested that pectin utilisation was is common among Bacteroides [25–27] and that neutral sugar-rich pectin is selectively metabolised to produce short-chain fatty acids and increase the beneficial *Bifidobacterial* population [28, 29]. Interestingly, pectin treatment indeed increased significantly the relative abundance of the *Bacteroidetes* group, along with *Streptococcus*, but the relative abundance of *Bifidobacteria* did not demonstrate significant change in our study. The relative abundance of *Clostridium*, *Clostridioides*, *Intestinibacter*, *Gemmiger*, *Muribaculum* and *Vibrio* decreased significantly. Previous research has revealed that *Clostridiaceae* was highly correlated with fat digestibility [30], so the decreased apparent digestibility coefficients of crude fat can be related to the decreased relative abundance of *Clostridium* and *Clostridioides* in the PEC group. These findings also suggest that feeding altered the composition and function of the colon microbiota, which could further serve as an important regulator affecting growth performance and gut health.

The colonic microbiomes encode a huge number of CAZymes to degrade polysaccharides beyond the capabilities of their host [31, 32]. In this study, these enzymes changed differently in the different diet groups. Thirty CAZyme families were altered significantly in the RPS diet group, 14 families changed significantly in the INU group and 35 families changed significantly in the PEC group. Among these CAZyme families, CBM26 and CBM41 decreased in all three diet groups compared with the CON group. They can be considered as families having a starch-binding domain (SBD) in the CAZyme database. An SBD is a special case of a carbohydrate-binding domain that has no enzymatic activity but can attach the catalytic domain to the carbohydrate substrate to hold it and process it at the active site, thereby bequeathing the enzymes the ability to bind onto raw starch [33, 34]. This may show that these three additives could prevent the degradation of raw starch. CBM61 increased in the PEC group compared with the CON group. Like all CBMs, CBM61 can increase the local concentration of enzymes on the substrate, thereby enhancing catalytic activity [35]. Indeed, most pectin-degrading microbes have their own stock of enzymes that include a variety of hydrolases and lyases that are able to degrade the arabinan, rhamnogalacturonan, polygalacturonan and galactan ‘domains’ in pectin, and the pectin-binding CBMs are limited. Moreover, although galactan could be recognised by CBM61, CBM61 binds to pectin with the greatest affinity, especially to samples containing the beta-1,4-galactan side chain component of pectin and to beta-1,4-galactotetraose, indicating its specificity for beta-1,4-linked galactose polymers [36]. This demonstrates that CBM61 is associated with the degradation of pectin. According to the correlation analysis between the CAZyme families and microbiota, *Streptococcus* was a main contributor of CBM61. The relative abundance of *Streptococcus* increased in the PEC group, which could also explain the increase of CBM61 in the PEC group.

Except for the CBM families, previous studies have shown that GHs and PLs are the two key types of CAZymes that degrade different substrates. GHs cleave bonds by the insertion of a water molecule [37, 38], while PLs cleave complex carbohydrates through an elimination mechanism [39]. CEs remove ester substituents from glycan chains to facilitate the action of GHs and PLs. GTs assemble complex carbohydrates from activated sugar donors [40]. AAs are mostly involved in the process of cellulose and lignin degradation [41]. They have little association with the digestion of dietary fibers. Given the foregoing, we further focused on the changes in GHs and PLs. GHs and PLs are the most prominent factors that influence the digestion of substrates. As previously reported, the GH13 and GH57 families have alpha-amylase enzyme specificity. Alpha-amylase represents the best known amylolytic enzyme. It catalyses the hydrolysis of alpha-1,4-glucosidic linkages in starch and related alpha-glucans with the retaining reaction mechanism [42, 43]. PL9 could produce two or more unsaturated galacturonates from pectic substrates, confirming that it is endo-pectate lyase [44]. Enzymes in the GH32 family could degrade inulin-type fructans with an endo-cleavage mode [45]. In the present study, consistent with the above-mentioned reports, GH13 increased in the RPS group and decreased in the INU and PEC groups. PL9 decreased in the INU group and increased in the RPS and PEC groups. Although some changes obtained were opposite those elicited by the studies mentioned earlier, GH57 decreased in the RPS and INU groups but increased in the PEC group to a very minimal extent. GH32 increased in the RPS and PEC groups but decreased in the INU group. These inconsistent changes could be ascribed to the comprehensive impacts of the many different types of enzyme subfamilies in these families.

More changes in the abundance of GHs occurred with the alteration of the diets in our study. Some of these changes warrant further research. The changes in the CAZymes corresponded to the different dominant bacteria. We also examined the relationship between the colonic microbiota and CAZymes. In the RPS group, the abundance of the GH14 and GH8 families downregulated while GH77 upregulated. GH14 was annotated as a beta-amylase (EC 3.2.1.2), which is a crucial exo-hydrolase that contributes to the complete degradation of starch into metabolisable or fermentable sugar [46]. GH8 was annotated as endohemicellulase; enzymes in the GH8 family could degrade hemicellulose and eliminate anti-nutritional effects [47]. They always show xylanase activity on heteroxylans from various sources [48]. The production of GH14 and GH8 had a negative correlation with *Parabacteroides*. Consistent with the results in human trials after consumption of resistant starches [49], the abundance of *Parabacteroides* increased in the RPS group in our study, the decrease of these enzymes may be related to the change in the bacteria, leading to a negative effect on nutrient digestion. We also found positive correlations of *Parabacteroides* to CAZyme GH88 in the RPS group. GH88 upregulated in the RPS group, so we supposed that GH88 played a role in utilizing raw potato starch. GH88 was annotated as d-4,5 unsaturated beta-glucuronyl hydrolase (EC 3.2.1.-). Glucuronyl hydrolase could convert conjugated bilirubin to unconjugated bilirubin, it had a major impact on liver function. However, the specific effect of this enzyme in the use of RPS is unclear now.

CAZyme GH77 increased in the RPS group. It was annotated as a debranching enzyme containing only one enzyme specificity of 4-alpha-glucanotransferase (EC 2.4.1.25) [50]. It is involved in maltose metabolism in microorganisms [51]. Since *Alloprevotella* and *Ruminococcus* contributed proportions of GH77, the enzyme increased along with the enrichment of *Alloprevotella* and *Ruminococcus* in the RPS group, making the digestion process of raw potato starch more efficient through maltose metabolism. The abundance of GH14, GH15, GH24, GH26, GH27, GH38 and GH101 downregulated in the INU group. All significantly changed GHs downregulated in the INU group, probably because of the negative effect of inulin on digestion. More CAZymes changes were found with the regulation of the PEC-enriched diet. The relative abundance of GH5, GH16, GH103 and GH102 increased while GH101 and GH112 decreased. The abundance of GH5 increased along with the increase of *Streptococcus* and *Prevotella*. GH5 was mainly annotated as endo-glucanase, which is the main ingredient of cellulase [52]. It is also involved in the hydrolysis of galactose. Some loops in GH5 enzymes could recognise galactosyl units [53]. The abundance of GH16 increased along with the increase of *Streptococcus* and *Mesorhizobium*, and the degradation of galactose was predicted to be initiated by GH16 [54]. This galactanase had a substrate specificity acting on galactooligosaccharides [55]. The abundance of GH103 decreased with the increase of *Clostridioides* and the decrease of *Vibrio*. The glycoside hydrolases of the GH103 family are in fact lytic transglycosylases of bacterial origin [56]. These enzymes cleave the beta-1,4 linkage between N-acetylmuramoyl and N-acetylglucosaminyl residues in peptidoglycan. The abundance of GH102 increased with the increase of *Intestinibacter*. Along with the decrease of *Gemmiger* bacteria, the abundance of GH101 and GH112 decreased in the PEC group. The GH101 family is made up of endo-alpha-N-acetylgalactosaminidases and their homologues [57]. GH112 almost contains phosphorylases such as lacto-N-biose phosphorylase, galacto-N-biose phosphorylase (EC 2.4.1.211) and D-galactosyl-1,4-L-rhamnose phosphorylase (EC 2.4.1.-). These CAZymes could affect the digestion of polysaccharides, thereby further modulating the digestion of different dietary fibers. Although we didn’t found the direct connection between the CAZymes and the growth performances of pigs, the digestion of dietary fiber probably have a negative impact on colon health, especially pectin. According to our previous study [58], compared with the CON group, pectin diet evidently damaged the colonic mucosal surface.

## Conclusion

In conclusion, the results of this exploratory study provided a comprehensive overview of the effects of different fibers on gut microbiota and CAZymes in pigs’ colons. In particular, it showed the function of *Streptococcus* along with CBM61 in the degradation of galactose in the PEC group, which will offer new insights into the impacts of the use of dietary fibers on animal and human health. This study unveiled the possibility of selectively regulating the abundance of colon microbiota by means of dietary fibers, thus obtaining a more in-depth understanding of the role of the different types of dietary fibers in regulating intestinal microbial metabolism.

## Methods

### Animal experiments and sample collection

Twenty-eight 35-day-old pigs (Duroc×Landrace×Large White) with similar body weight (mean ± SEM, 8.79 ± 0.09 kg) were randomly designated into four groups, with each group consisting of seven replicates (pens) with one pig per pen. The pigs in the four groups were fed with control (CON) diet (a corn-soybean based diet), inulin (INU), raw potato starch (RPS) or pectin (PEC) enriched diets, respectively. Based on the literature, the addition of different dietary fibers ranging from 3 to 10% (w/w) is reasonable. In this study, to conduct a comparison of the effects of the three dietary fibres under uniform conditions, inulin, raw potato starch or pectin were used to replace 8% (w/w) corn starch in the CON diet (Additional File 4). The trial lasted for 40 days. The pigs had unlimited access to feed and water throughout the experimental period. At the age of 76 days, all pigs were anaesthetised and euthanised with a jugular vein injection of 4% sodium pentobarbital solution (40 mg/kg body weight) after a 12-h fast. Proximal colonic luminal digesta samples were collected, snap-frozen using liquid nitrogen and stored at − 80 °C until further analysis.

### RNA extraction and metatranscriptomic sequencing

Total RNA was extracted from each proximal colonic luminal digesta sample of pigs with TRIzol reagent (Invitrogen, CA, USA) in accordance with the manufacturer’s protocols and subjected to DNase I (TaKara, Dalian, China) digestion to remove contaminating DNA. Given that there were seven replicates in each group, four biological replicates were randomly selected for the RNA-Seq to reduce the experimental expense. Then, the total RNA quantity and purity were analysed using Bioanalyzer 2100 and RNA 6000 Nano Lab Chip Kit (Agilent, CA, USA) with RIN number > 7.0. A total of 5 μg of RNA per sample was processed for rRNA depletion using a Ribo-Zero™ Magnetic kit (G+/G-Bacteria). A high-quality RNA sample (optical density (OD) 260/280 = 1.8–2.2) was used to construct the sequencing library. Following the TruSeq RNA preparation kit from Illumina (San Diego, CA, USA), the RNA was divided into small pieces and used as a template for cDNA synthesis. A polymerase chain reaction (PCR) solution containing a mixture of dATP, dGTP, dCTP and dUTP was used, and the PCR reaction was amplified for 15 cycles. In brief, libraries were size-selected for cDNA target fragments of 200–300 bp on 2% certified low-range ultra agarose, followed by PCR amplification using Phusion DNA polymerase (NEB). After quantification with TBS380, the paired-end libraries were sequenced by Shanghai Biozeron Biotechnology Co. Ltd. (Shanghai, China), and the read length was Illumina HiSeq PE 2 × 150 bp. The raw sequence reads were submitted to the NCBI Sequence Reads Archive (SRA) under Submission Bioproject ID: PRJNA693413.

### Metatranscriptome data analysis

The raw sequence reads were subjected to filtering of host reads, adapter sequences or poly-N and low-quality (Q < 20) sequences. (http://bio-bwa.sourceforge.net; https://github.com/jstjohn/SeqPrep;https://github.com/najoshi/sickle). The Q20, Q30 and GC contents of the quality-filtered data were calculated. Ribosomal RNA sequences were removed through comparisons with the NCBI rRNA, tRNA and SILVA databases. The remaining quality-filtered sequence reads were assembled de novo into transcripts using Megahit (https://github.com/voutcn/megahit) with the default parameters. Then, the transcripts of all 16 samples were combined and clustered into unique classes with CD-HIT-EST at 95% identity. After the assembly and clustering of the transcripts, the longest sequence of each class was treated as a unigene. The average total reads of each sample was 96,396,981 in raw data, and the average total reads of each sample was 88,577,377 in clean data. To avoid deviations caused by different sequencing depths among the samples, the total hit reads in the proximal colonic luminal digesta samples were normalised to the size of the sequencing data. The normalization as follows: calculate the ppm abundance according to the reads abundance, ppm = {[(reads/contig)/total reads]/total abundance}*1,000,000, and the total abundance of each sample was 1,000,000. The expression of each unigene was evaluated as parts per million (PPM) [59]. ‘PPM’ refers to a certain gene read in one million metatranscriptomic sequencing reads. Then, differentially expressed genes (DEGs) between two different groups were identified according to the PPM. Genes with altered expression (fold change > 2 or < 0.5; FDR < 0.05) were selected for further study.

### Taxonomic annotation of unigenes

BLASTP (BLAST version 2.2.28+, http: //blast.ncbi.nlm.nih.gov/Blast.cgi) was used to annotate the unigenes by comparing the genes against the NR database (e-value <1e− 5). The abundance of each taxonomic level according to the sum of the corresponding gene abundance in each sample was calculated and the abundance profile at the corresponding taxonomic level was measured. Then, the PCoA of these samples were determined based on the Bray-Curtis distances.

### Functional annotation of CAZymes

CAZyme functional annotation was carried out using hmmscan (http://hmmer.janelia.org/search/hmmscan/)(e-value <1e− 5), and then the annotation of the CAZyme corresponding to the gene was obtained.

### Analysis of correlations between bacterial genera and CAZymes

The correlations between the observed microbial taxa (bacterial genera) and CAZymes were explored using Spearman’s rank correlation. Significant relationships (coefficient [p] of > 0.5 or < − 0.5 and *p*-value < 0.05) between the observed microbial taxa and CAZymes were selected for further study. Cytoscape (version 3.2.1) [60] was utilised to visualise the network of correlations between the bacteria and CAZyme families.

### Analysis of microbiota distributed to the CAZymes

The distributions of microbiota to the CAZymes were visualised via Circos. The contributions of different bacteria to certain enzymes were evaluated by analysing the origin of the enzymes. Then, the microbiota were ordered according to the distribution. The top 10 and 15 genera were respectively selected. The influence of the bacteria on the digestion of dietary fibers will be analysed in follow-up studies.

## Supplementary Information

**Additional file 1.** The changed phyla in the dietary fiber groups compared to the CON group. CON, a control diet; RPS, a raw potato starch enriched diet; INU, an inulin enriched diet; PEC, a pectin enriched diet.

**Additional file 2.** The CAZymes changes in the dietary fiber groups compared to the CON group. CON, a control diet; RPS, a raw potato starch enriched diet; INU, an inulin enriched diet; PEC, a pectin enriched diet.

**Additional file 3.** The network of correlations between bacteria and CAZyme classes changed significantly by the dietary fiber. Green lines represent positive correlations and red lines represent negative correlations

**Additional file 4.** Composition and analyzed nutrient contents of experimental diets (as-fed basis).

## Abbreviations

CAZymes: Carbohydrate active enzymes
CON: Control group
RPS: Raw potato starch group
INU: Inulin group
PEC: Pectin group
FDR: False discovery rate
GHs: Glycoside hydrolases
PCoA: Principal coordinate analysis
AAs: Auxiliary activities
CBMs: Carbohydrate-binding modules
CEs: Carbohydrate esterases
GTs: Glycosyl transferases
PLs: Polysaccharide lyases
SBD: Starch-binding domain

## References

[1] Grabitske HA, Slavin JL (2008). Low-digestible carbohydrates in practice. J Am Diet Assoc.

[2] Thebaudin JY, Lefebvre AC, Harrington M, Bourgeois CM (1997). Dietary fibers: nutritional and technological interest. Trends Food Sci Tech.

[3] Metzler BU, Mosenthin R (2008). A review of interactions between dietary fiber and the gastrointestinal microbiota and their consequences on intestinal phosphorus metabolism in growing pigs. Asian Austral J Anim.

[4] Tap J, Furet J, Bensaada M, Philippe C, Roth H, Rabot S (2015). Gut microbiota richness promotes its stability upon increased dietary fiber intake in healthy adults. Environ Microbiol.

[5] El Kaoutari A, Armougom F, Gordon JI, Raoult D, Henrissat B (2013). The abundance and variety of carbohydrate-active enzymes in the human gut microbiota. Nat Rev Microbiol.

[6] Lattimer JM, Haub MD (2010). Effects of dietary Fiber and its components on metabolic health. Nutrients..

[7] Kawakami S, Han KH, Araki T, Ohba K, Wakabayashi T, Shimada K (2017). Potato powders prepared by successive cooking-process depending on resistant starch content affect the intestinal fermentation in rats. Biosci Biotechnol Biochem.

[8] Dumont MG, Pommerenke B, Casper P (2013). Using stable isotope probing to obtain a targeted metatranscriptome of aerobic methanotrophs in lake sediment. Environ Microbiol Rep.

[9] Wang J, Xu R, Xiang X, Su Y, Zhu W. Transcriptomic and metabolomic responses in the livers of pigs to diets containing different non-starchy polysaccharides. J Funct Foods. 2020;64:103590.

[10] Eberhard M, Hennig U, Kuhla S, Brunner RM, Kleessen B, Metges CC (2007). Effect of inulin supplementation on selected gastric, duodenal, and caecal microbiota and short chain fatty acid pattern in growing piglets. Arch Anim Nutr.

[11] Higgins JA, Brown MA, Storlien LH. Consumption of resistant starch decreases postprandial lipogenesis in white adipose tissue of the rat. Nutr J. 2006;5(1):25. 10.1186/1475-2891-5-25 PMC1618391 16987425

[12] Ferreira-Lazarte A, Fernandez J, Gallego-Lobillo P, Villar CJ, Lombo F, Moreno FJ, et al. Behaviour of citrus pectin and modified citrus pectin in an azoxymethane/dextran sodium sulfate (AOM/DSS)-induced rat colorectal carcinogenesis model. Int J Biol Macromol. 2020;167. 10.1016/j.ijbiomac.2020.11.089 33202274

[13] Bohmer BM, Branner GR, Roth-Maier DA (2005). Precaecal and faecal digestibility of inulin (DP 10–12) or an inulin/*Enterococcus faecium* mix and effects on nutrient digestibility and microbial gut flora. J Anim Physiol Anim Nutr (Berl).

[14] Wang JF, Jensen BB, Jorgensen H, Li DF, Lindberg JE. Ileal and total tract digestibility, and protein and fat balance in pigs fed rice with addition of potato starch, sugar beet pulp or wheat bran. Anim Feed Sci Tech. 2002;102(1–4):125–36.

[15] Martinez-Puig D, Perez JF, Castillo M, Andaluz A, Anguita M, Morales J (2003). Consumption of raw potato starch increases colon length and fecal excretion of purine bases in growing pigs. J Nutr.

[16] Alzueta C, Rodriguez ML, Ortiz LT, Rebole A, Trevino J (2010). Effects of inulin on growth performance, nutrient digestibility and metabolisable energy in broiler chickens. Br Poult Sci.

[17] Silva VK, Morita VDS, Boleli IC (2013). Effect of pectin extracted from citrus pulp on digesta characteristics and nutrient digestibility in broilers chickens. Rev Bras Zootecn.

[18] Beloshapka AN, Cross TL, Swanson KS. Graded dietary resistant starch concentrations on apparent total tract macronutrient digestibility and fecal fermentative End-Products and microbial populations of healthy adult dogs. J Anim Sci. 2020;1:1. 10.1093/jas/skaa409 PMC7819633 33373446

[19] Zhang X, Hou Z, Xu B, Xie C, Wang Z, Yu X, et al. Dietary supplementation of epsilon-Polylysine beneficially affects ileal microbiota structure and function in ningxiang pigs. Front Microbiol. 2020;11:544097. 10.3389/fmicb.2020.544097 PMC7702972 33312165

[20] Bang S, Lee E, Song E, Nam Y, Seo M, Kim H (2019). Effect of raw potato starch on the gut microbiome and metabolome in mice. Int J Biol Macromol.

[21] Zhang S, Yang J, Henning SM, Lee R, Hsu M, Grojean E, Pisegna R, Ly A, Heber D, Li Z (2017). Dietary pomegranate extract and inulin affect gut microbiome differentially in mice fed an obesogenic diet. Anaerobe..

[22] Le Bastard Q, Chapelet G, Javaudin F, Lepelletier D, Batard E, Montassier E. The effects of inulin on gut microbial composition: a systematic review of evidence from human studies. Eur J Clin Microbiol Infect Dis. 2020;39:3. 10.1007/s10096-019-03721-w 31707507

[23] Anzawa D, Mawatari T, Tanaka Y, Yamamoto M, Genda T, Takahashi S (2019). Effects of synbiotics containing Bifidobacterium animalis subsp. Lactis GCL2505 and inulin on intestinal bifidobacteria: a randomized, placebo-controlled, crossover study. Food Sci Nutr.

[24] Zhang Q, Yu H, Xiao X, Hu L, Xin F, Yu X. Inulin-type fructan improves diabetic phenotype and gut microbiota profiles in rats. Peerj. 2018;6(3):e4446. 10.7717/peerj.4446 PMC5835350 29507837

[25] Dongowski G, Lorenz A, Anger H (2000). Degradation of pectins with different degrees of esterification by Bacteroides thetaiotaomicron isolated from human gut flora. Appl Environ Microb.

[26] Chung WSF, Meijerink M, Zeuner B, Holck J, Louis P, Meyer AS, et al. Prebiotic potential of pectin and pectic oligosaccharides to promote antiinflammatory commensal bacteria in the human colon. Fems Microbiol Ecol. 2017;93:fix127. 10.1093/femsec/fix127 29029078

[27] Luis AS, Briggs J, Zhang X, Farnell B, Ndeh D, Labourel A (2018). Dietary pectic glycans are degraded by coordinated enzyme pathways in human colonic Bacteroides. Nat Microbiol.

[28] Onumpai C, Kolida S, Bonnin E, Rastall RA (2011). Microbial utilization and selectivity of pectin fractions with various structures. Appl Environ Microb..

[29] Gomez B, Gullon B, Yanez R, Schols H, Alonso JL (2016). Prebiotic potential of pectins and pectic oligosaccharides derived from lemon peel wastes and sugar beet pulp: a comparative evaluation. J Funct Foods.

[30] Bermingham EN, Maclean P, Thomas DG, Cave NJ, Young W. Key bacterial families (Clostridiaceae, Erysipelotrichaceae and Bacteroidaceae) are related to the digestion of protein and energy in dogs. Peerj. 2017;5:e3019. 10.7717/peerj.3019 PMC5337088 28265505

[31] Hehemann J, Kelly AG, Pudlo NA, Martens EC, Boraston AB (2012). Bacteria of the human gut microbiome catabolize red seaweed glycans with carbohydrate-active enzyme updates from extrinsic microbes. P Natl Acad Sci Usa.

[32] El Kaoutari A, Armougom F, Leroy Q, Vialettes B, Million M, Raoult D, et al. Development and validation of a microarray for the investigation of the CAZymes encoded by the human gut microbiome. Plos One. 2013:e8403312. 10.1371/journal.pone.0084033 PMC3877134 24391873

[33] Gilbert HJ (2003). How carbohydrate binding modules overcome ligand complexity. Structure..

[34] Janecek S, Marecek F, MacGregor EA, Svensson B (2019). Starch-binding domains as CBM families-history, occurrence, structure, function and evolution. Biotechnol Adv.

[35] Rooijakkers B, Arola S, Velagapudi R, Linder MB. Different effects of carbohydrate binding modules on the viscoelasticity of nanocellulose gels. Biochem Biophys Rep. 2020;22:100766. 10.1016/j.bbrep.2020.100766 PMC7176825 32337376

[36] Cid M, Pedersen HL, Kaneko S, Coutinho PM, Henrissat B, Willats WG (2010). Recognition of the helical structure of beta-1,4-galactan by a new family of carbohydrate-binding modules. J Biol Chem.

[37] Henrissat B (1991). A classification of glycosyl hydrolases based on amino-acid-sequence similarities. Biochem J.

[38] Koropatkin NM, Cameron EA, Martens EC (2012). How glycan metabolism shapes the human gut microbiota. Nat Rev Microbiol..

[39] Lombard V, Bernard T, Rancurel C, Brumer H, Coutinho PM, Henrissat B (2010). A hierarchical classification of polysaccharide lyases for glycogenomics. Biochem J.

[40] Lairson LL, Henrissat B, Davies GJ, Withers SG (2008). Glycosyltransferases: structures, functions, and mechanisms. Annu Rev Biochem.

[41] Levasseur A, Drula E, Lombard V, Coutinho PM, Henrissat B. Expansion of the enzymatic repertoire of the CAZy database to integrate auxiliary redox enzymes. Biotechnol Biofuels. 2013;6(1):41. 10.1186/1754-6834-6-41 PMC3620520 23514094

[42] Janecek S, Kuchtova A, Petrovicova S (2015). A novel GH13 subfamily of alpha-amylases with a pair of tryptophans in the helix alpha 3 of the catalytic TIM-barrel, the LPDlx signature in the conserved sequence region V and a conserved aromatic motif at the C-terminus. Biologia..

[43] Janecek S, Svensson B, MacGregor EA (2014). Alpha-amylase: an enzyme specificity found in various families of glycoside hydrolases. Cell Mol Life Sci.

[44] Chakraborty S, Fernandes VO, Dias FMV, Prates JAM, Ferreira LMA, Fontes CMGA, et al. Role of Pectinolytic Enzymes Identified in Clostridium thermocellum Cellulosome. Plos One. 2015;10(2):e01167872. 10.1371/journal.pone.0116787 PMC4319962 25658912

[45] Pouyez J, Mayard A, Vandamme A, Roussel G, Perpete EA, Wouters J (2012). First crystal structure of an endo-inulinase, INU2, from Aspergillus ficuum: discovery of an extra-pocket in the catalytic domain responsible for its endo-activity. Biochimie..

[46] Ben HN. Prediction and analysis of GH14 family beta-amylases in oat seedling extract: structure and function insights using in silico approaches. Int J Biol Macromol. 2019;125:361–9. 10.1016/j.ijbiomac.2018.12.065 30528996

[47] Bohra V, Dafale NA, Purohit HJ (2019). Understanding the alteration in rumen microbiome and CAZymes profile with diet and host through comparative metagenomic approach. Arch Microbiol.

[48] Ray S, Vigouroux J, Bouder A, Allami MF, Geairon A, Fanuel M (2019). Functional exploration of Pseudoalteromonas atlantica as a source of hemicellulose-active enzymes: evidence for a GH8 xylanase with unusual mode of action. Enzyme Microb Tech.

[49] Upadhyaya B, Mccormack L, Fardin-kia AR, Juenemann R. Impact of dietary resistant starch type 4 on human gut microbiota and immunometabolic functions. Sci Rep. 2016;6:28797. 10.1038/srep28797 PMC4928084 27356770

[50] Kuchtova A, Janecek S (2015). In silico analysis of family GH77 with focus on amylomaltases from borreliae and disproportionating enzymes DPE2 from plants and bacteria. Biochim Biophys Acta.

[51] Kaper T, Leemhuis H, Uitdehaag JCM, van der Veen BA, Dijkstra BW, van der Maarel MJEC (2007). Identification of acceptor substrate binding subsites+2 and+3 in the amylomaltase from Thermus thermophilus HB8. Biochemistry-Us..

[52] Park JI, Kent MS, Datta S, Holmes BM, Huang Z, Simmons BA (2011). Enzymatic hydrolysis of cellulose by the cellobiohydrolase domain of CelB from the hyperthermophilic bacterium Caldicellulosiruptor saccharolyticus. Bioresour Technol.

[53] Kumagai Y, Yamashita K, Tagami T, Uraji M, Wan K, Okuyama M (2015). The loop structure of Actinomycete glycoside hydrolase family 5 mannanases governs substrate recognition. FEBS J.

[54] Kwon GH, Kwon MJ, Park JE, Kim YH. Whole genome sequence of a freshwater agar-degrading bacterium Cellvibrio sp. KY-GH-1. Biotechnol Rep (Amst). 2019;23:e346. 10.1016/j.btre.2019.e00346 PMC6535462 31193527

[55] Kalomoiri P, Holck J, Coulomb L, Boos I, Enemark-Rasmussen K, Spodsberg N, Monrad RN, Clausen MH (2019). Substrate specificity of novel GH16 endo-beta-(1-->3)-galactanases acting on linear and branched beta-(1-->3)-galactooligosaccharides. J Biotechnol.

[56] Blackburn NT, Clarke AJ (2001). Identification of four families of peptidoglycan lytic transglycosylases. J Mol Evol.

[57] Naumoff DG (2010). Gh101 family of glycoside hydrolases: subfamily structure and evolutionary connections with other families. J Bioinf Comput Biol.

[58] Xu R, Lu Y, Wang J, Liu J, Su Y, Zhu W (2019). Effects of the different dietary fibers on luminal microbiota composition and mucosal gene expression in pig colons. J Funct Foods.

[59] Chen B, Yang Y, Liang X, Yu K, Zhang T, Li X (2013). Metagenomic profiles of antibiotic resistance genes (ARGs) between human impacted estuary and deep ocean sediments. Environ Sci Technol.

[60] Shannon P, Markiel A, Ozier O, Baliga NS, Wang JT, Ramage D (2003). Cytoscape: a software environment for integrated models of biomolecular interaction networks. Genome Res.

