# Supplementary material for: Metatranscriptomic analysis of colonic microbiota’s functional response to different dietary fibers in growing pigs
Source: Anim Microbiome. 2021 Jul 3;3:45. doi: 10.1186/s42523-021-00108-1 (PMC8254964; doi:10.1186/s42523-021-00108-1)
Supplement: Supplementary file 1 — Additional file 1. The changed phyla in the dietary fiber groups compared to the CON group. CON, a control diet; RPS, a raw potato starch enriched diet; INU, an inulin enriched diet; PEC, a pectin enriched diet. [file 42523_2021_108_MOESM1_ESM.docx]

**Additional File 1**

**The changed phyla in the dietary fiber groups compared to the CON group.** CON, a control diet; RPS, a raw potato starch enriched diet; INU, an inulin enriched diet; PEC, a pectin enriched diet.

**
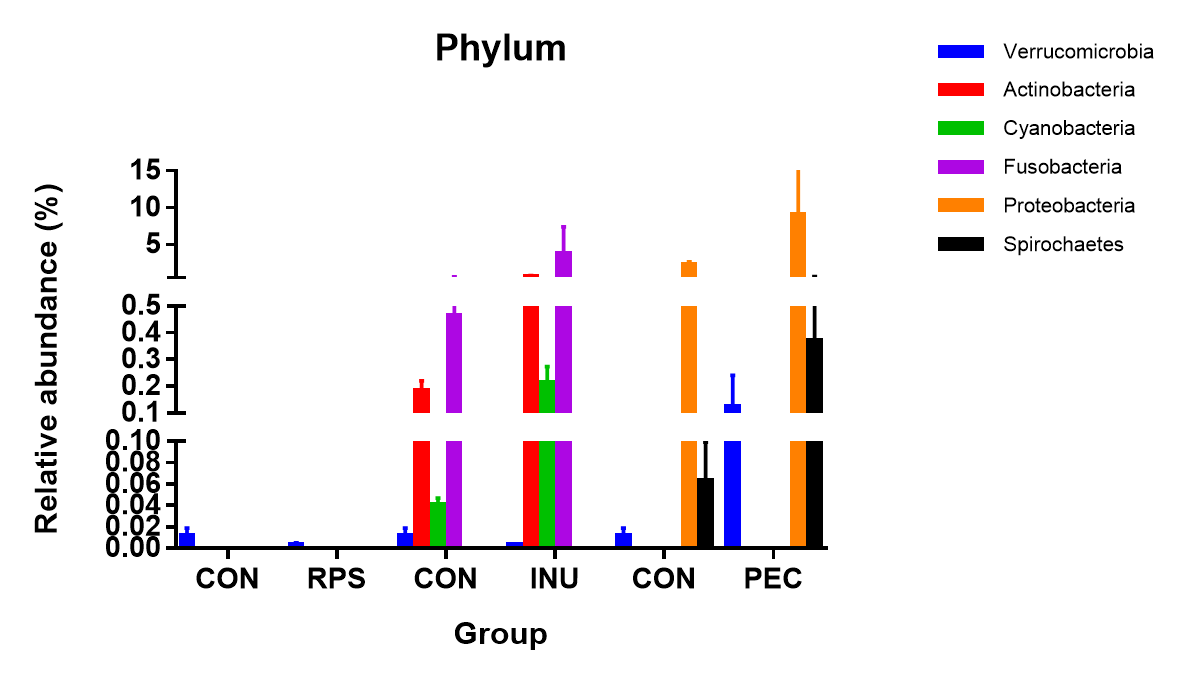
**
