# Supplementary material for: Metatranscriptomic analysis of colonic microbiota’s functional response to different dietary fibers in growing pigs
Source: Anim Microbiome. 2021 Jul 3;3:45. doi: 10.1186/s42523-021-00108-1 (PMC8254964; doi:10.1186/s42523-021-00108-1)

**Additional File 3**

**The network of correlations between bacteria and CAZyme classes changed significantly by the dietary fiber.** Green lines represent positive correlations and red lines represent negative correlations.


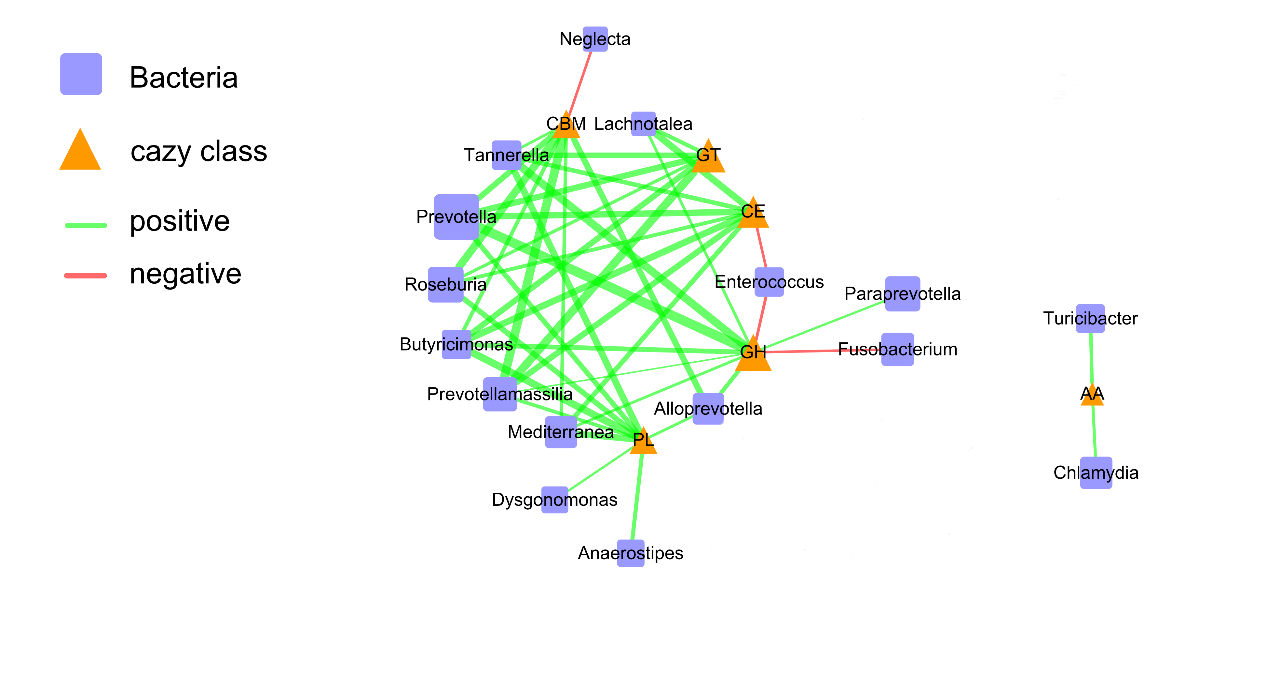

Supplement: Supplementary file 3 — Additional file 3. The network of correlations between bacteria and CAZyme classes changed significantly by the dietary fiber. Green lines represent positive correlations and red lines represent negative correlations [file 42523_2021_108_MOESM3_ESM.docx]
