# Supplementary material for: Metatranscriptomic analysis of colonic microbiota’s functional response to different dietary fibers in growing pigs
Source: Anim Microbiome. 2021 Jul 3;3:45. doi: 10.1186/s42523-021-00108-1 (PMC8254964; doi:10.1186/s42523-021-00108-1)
Supplement: Supplementary file 4 — Additional file 4. Composition and analyzed nutrient contents of experimental diets (as-fed basis). [file 42523_2021_108_MOESM4_ESM.docx]

**Additional File 4**

Composition and analyzed nutrient contents of experimental diets (as-fed basis).

| Diet | CON | RPS | INU | PEC |
| --- | --- | --- | --- | --- |
| Ingredients (g/kg) | | | | |
| Corn starch | 80.0 | 0 | 0 | 0 |
| Raw potato starch | 0 | 80.0 | 0 | 0 |
| Inulin | 0 | 0 | 80.0 | 0 |
| Pectin | 0 | 0 | 0 | 80.0 |
| Corn | 518 | 518 | 518 | 518 |
| Wheat bran | 90.0 | 90.0 | 90.0 | 90.0 |
| Soybean meal | 280 | 280 | 280 | 280 |
| Dicalcium phosphate | 9.00 | 9.00 | 9.00 | 9.00 |
| Limestone | 9.00 | 9.00 | 9.00 | 9.00 |
| Salt | 3.00 | 3.00 | 3.00 | 3.00 |
| Vitamin and mineral premix^a^ | 10.0 | 10.0 | 10.0 | 10.0 |
| L-Lysine | 1.00 | 1.00 | 1.00 | 1.00 |
| Nutrient analysis (g/kg) | | | | |
| CP | 19.12 | 19.12 | 19.12 | 19.12 |
| Ash | 7.97 | 7.97 | 7.97 | 7.97 |
| CF | 3.11 | 3.28 | 3.91 | 3.31 |

^a^ This mineral and vitamin premix (1%) supplies per kg diet as follows: VA 11 000 IU, VD3 1 000 IU, VE 16 IU, VK1 1mg, VB1 0.6 mg, VB2 0.6 mg, d-pantothenic acid 6 mg, nicotinic acid 10 mg, VB12 0.03 mg, folic acid 0.8 mg, VB6 1.5 mg, choline 800 mg, Fe 165 mg, Zn 165 mg, Cu 16.5 mg, Mn 30 mg, Co 0.15 mg, I 0.25 mg, Se 0.25 mg.
